# Corrigendum: Mapping and validating a point neuron model on intel's neuromorphic hardware Loihi

**DOI:** 10.3389/fninf.2022.1023486

**Published:** 2022-09-14

**Authors:** Srijanie Dey, Alexander Dimitrov

**Affiliations:** Department of Mathematics, Washington State University, Vancouver, WA, United States

**Keywords:** neuromorphic computing, LIF models, neural simulations, validation, performance analysis

In the published article, there was an error in Figure 7 and Figure 9 as published. The units for the Root Mean Square Error (RMSE) in both figures were in mV and pA (per run, about 500ms), but should have been in mV/ms and pA/ms as per aforementioned results. The revised figures with the corrected units as mV/ms and pA/ms appear below.

**Figure 7:**
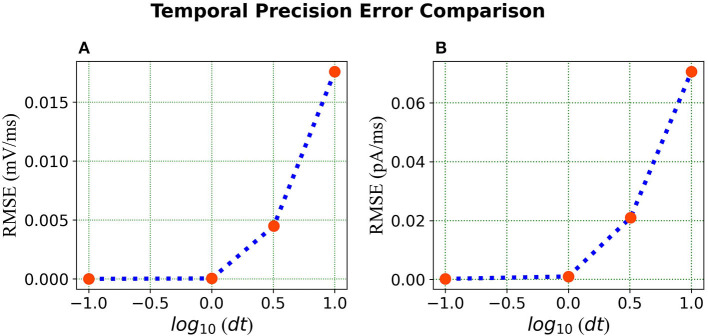
Error comparison for different temporal precisions—**(A)** Membrane potential error. **(B)** Current error. In both panels, the RMSE for the corresponding state is plotted against the *log* of the temporal precision *dt*.

**Figure 9:**
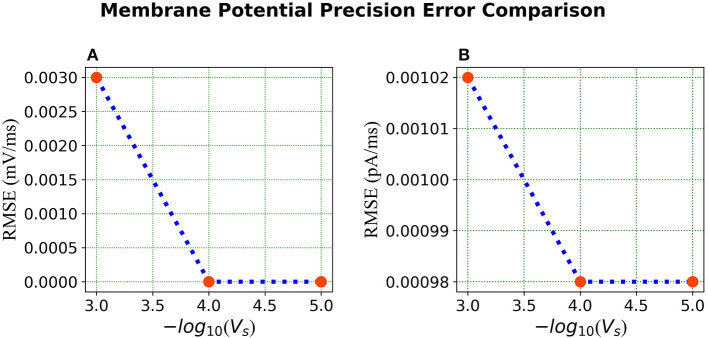
Error comparison for different membrane potential precisions—**(A)** Membrane potential error. **(B)** Current error. In both panels, the RMSE for the corresponding state is plotted against the –*log* of the voltage scale *V*_*s*_.

In the published article, there was also an error in **Results, Simulation of Different Neuron Classes**, Paragraph 4. The range of the bias mantissa is incorrectly stated as [2^−12^, 2^12^] when it should be [-2^12^, 2^12^]. A correction has been made to the paragraph below:

“We reiterate here that Loihi imposes certain bit constraints on the parameters. For instance, membrane potential threshold ranges from 0 to ± 2^23^, membrane time constant allows 0 to 2^12^ bits. The membrane capacitance is integrated with bias current (Equation 18) with bias mantissa allowed a range between [−2^12^, 2^12^] and bias exponent a range between [0, 7]. Thus, a good range of parameters can be mapped well into Loihi and a limit to the “exactness” can be attributed to the low-fixed-precision nature of Loihi as most state and configuration variables are in the range of 8–24 bits”.

The authors apologize for these errors and state that this does not change the scientific conclusions of the article in any way. The original article has been updated.

## Publisher's note

All claims expressed in this article are solely those of the authors and do not necessarily represent those of their affiliated organizations, or those of the publisher, the editors and the reviewers. Any product that may be evaluated in this article, or claim that may be made by its manufacturer, is not guaranteed or endorsed by the publisher.

